# Speckle tracking echocardiography in patients with septic shock: a case control study (SPECKSS)

**DOI:** 10.1186/s13054-016-1327-0

**Published:** 2016-05-14

**Authors:** Pauline Yeung Ng, Wai Ching Sin, Andrew Kei-Yan Ng, Wai Ming Chan

**Affiliations:** Department of Adult Intensive Care, Queen Mary Hospital, 102 Pokfulam Road, Hong Kong, China; Cardiac Medical Unit, Grantham Hospital, 125 Wong Chuk Hang Road, Hong Kong, China

**Keywords:** Sepsis, Septic shock, Septic cardiomyopathy, Sepsis-induced myocardial dysfunction, Speckle tracking echocardiography, Speckle tracking imaging, Strain, Global longitudinal strain

## Abstract

**Background:**

Sepsis-induced myocardial dysfunction is a well-recognized condition and confers worse outcomes in septic patients. Echocardiographic assessment by conventional parameters such as left ventricular ejection fraction (LVEF) is often affected by ongoing changes in preload and afterload conditions. Novel echocardiographic technologies such as speckle tracking echocardiography (STE) have evolved for direct assessment of the myocardial function. We investigate the measurement of myocardial strain by speckle tracking echocardiography for the diagnosis of sepsis-induced myocardial dysfunction.

**Methods:**

This is a case-control study at a university-affiliated medical intensive care unit. Consecutive adult medical patients admitted with a diagnosis of septic shock were included. Patients with other causes of myocardial dysfunction were excluded. They were compared to age-matched, gender-matched, and cardiovascular risk-factor-matched controls, who were admitted to hospital for sepsis but did not develop septic shock. Transthoracic echocardiography was performed on all patients within 24 hours of diagnosis, and a reassessment echocardiogram was performed in the study group of patients upon recovery.

**Results:**

Patients with septic shock (*n* = 33) (study group) and 29 matched patients with sepsis but no septic shock (control group) were recruited. The mean sequential organ failure assessment (SOFA) score for the study and control groups were 10.2 and 1.6, respectively (*P* < 0.001). In patients with septic shock, the mean arterial pressure was lower (76 mmHg vs 82 mmHg, *P* = 0.032), and the heart rate was higher (99 bpm vs 86 bpm, *P* = 0.008). The cardiac output (5.9 L/min vs 5.5 L/min, *P* = 0.401) and systemic vascular resistance (1090 dynes•sec/cm^5^ vs 1194 dynes•sec/cm^5^, *P* = 0.303) were similar. The study group had a greater degree of myocardial dysfunction measured by global longitudinal strain (GLS) (–14.5 % vs –18.3 %, *P* <0.001), and the myocardial strain differed upon diagnosis and recovery (–14.5 % vs –16.0 %, *P* = 0.010). Conventional echocardiographic measurements such as LVEF (59 % in the study group vs 61 % in the control group, *P* = 0.169) did not differ between the two groups.

**Conclusion:**

Speckle tracking echocardiography can detect significant left ventricular impairment in patients with septic shock, which was not otherwise detectable by conventional echocardiography. The reversible nature of myocardial dysfunction in sepsis was also demonstrable. This echocardiographic technique is useful in the diagnosis and monitoring of sepsis-induced myocardial dysfunction.

## Background

Sepsis is one of the commonest conditions necessitating patient admission to the intensive care unit, and is associated with a myriad of organ dysfunctions. Sepsis-induced myocardial depression, or septic cardiomyopathy, is a well-recognized organ-specific manifestation in sepsis. The condition refers to depressed myocardial function during sepsis, which is fully reversible upon recovery. Septic cardiomyopathy was first described in 1984 by Parker et al, using the technique of radionuclide ventriculography, showing a significant incidence of impaired left ventricular ejection fraction (LVEF) of less than 40 % in patients with septic shock [1]. However, in more than 30 years since the condition was described, the diagnosis of septic cardiomyopathy has remained challenging. The difficulty in establishing a diagnosis lies in the lack of a test with adequate sensitivity and specificity for bedside diagnosis and serial monitoring of the myocardial dysfunction.

Two-dimensional transthoracic echocardiography is nowadays one of the first line investigations in patients with septic shock because of its accessibility and non-invasive nature. It is useful in ruling out other causes of hypotension such as cardiogenic and obstructive shock. In sepsis, however, assessment of myocardial function by conventional echocardiographic parameters such as left ventricular ejection fraction (LVEF) is affected to a large degree by ongoing changes in preload and afterload conditions. Other methods of quantifying the left and right ventricular function, such as the use of Doppler-derived indices in the calculation of the myocardial performance index (MPI) [2], and the use of tissue Doppler imaging (TDI) in measuring myocardial velocities [3, 4], are limited by being angle-dependent and less reproducible.

Speckle tracking echocardiography (STE) was first described in 2004 as a method of non-Doppler-based and angle-independent measurement of left ventricular function [5, 6]. Based on a semi-automated algorithm that tracks the displacement of acoustic “speckles” in the myocardium, the change in length of myocardial segments are measured. Compared to LVEF, STE is affected to a much lesser degree by changes in ventricular loading conditions, myocardial compliance, and afterload properties because it measures myocardial deformation directly. The commonest unit of measurement in STE is strain, defined as the change in the length of myocardial fiber at end-systole compared to its original length at end-diastole, expressed as a percentage. Strain can be measured in the longitudinal, radial, and circumferential directions. Global longitudinal strain (GLS), in averaging the longitudinal strain for all 17 myocardial segments, has been validated as the most consistently reproducible measurement [7]. The American Society of Echocardiography suggests a peak GLS in the range of –20 % be expected in a healthy person [8]. A recent joint initiative by the European Association of Cardiovascular Imaging, the American Society of Echocardiography, and the ultrasound industry has found strain measurements to be robust and reproducible, outperforming most conventional echocardiographic parameters [9].

In this study, we piloted the application of speckle tracking echocardiography in sepsis-induced myocardial dysfunction. We investigated the measurement of myocardial strain to describe the incidence and clinical progression of this clinical entity. We hypothesized that myocardial depression, measured by STE, is present in intensive care patients with septic shock, and is reversible upon recovery.

## Methods

This is a single center, case-control study conducted at a university-affiliated tertiary care adult intensive care unit. The study protocol (Institutional Review Board (IRB) reference number: UW 13-592) was approved by the IRB of The University of Hong Kong (HKU) and Hospital Authority Hong Kong West Cluster (HA HKW). Written informed consent was provided by all patients, and if the patient's fitness to consent was impaired at the time of recruitment, written consent was obtained from the patient’s next of kin.

### Definitions

Definitions laid out by the International Sepsis Definition Conference [10] were adopted. Sepsis is defined as the clinical syndrome of presence of both infection and a systemic inflammatory response. Septic shock refers to a state of hypotension with systolic arterial pressure below 90 mmHg, mean arterial pressure below 60 mmHg, or a reduction in systolic blood pressure more than 40 mmHg from baseline, despite adequate fluid resuscitation and in the absence of other causes of hypotension. Severity of illness was assessed by the sequential organ failure assessment (SOFA) score [11] on the day of admission to the intensive care unit (study group) or medical ward (control group).

### Study participants

Consecutive adult medical patients admitted to the intensive care unit were screened and recruited (study group) if they met the following criteria: (1) were aged 18 years or older, (2) had clinical symptoms suggestive of sepsis, and (3) developed septic shock requiring the use of inotropes or vasopressors. They were compared to a group of age-matched, gender-matched and cardiovascular risk-factor-matched controls (control group), who were admitted to hospital for sepsis but did not develop septic shock.

### Exclusion criteria

Patients were excluded if they met one of the following criteria: (1) active diagnoses directly relating to myocardial dysfunction, such as acute myocardial infarction, myocarditis, unstable arrhythmia, and post-cardiopulmonary resuscitation status; (2) significant underlying cardiac conditions, such as congenital heart disease, valvular heart disease, and cardiomyopathy; (3) informed consent could not be obtained; and (4) rapid clinical deterioration did not permit timely completion of the echocardiograms.

### Two-dimensional transthoracic echocardiography study

Two serial transthoracic echocardiographic examinations were performed in the study group of patients, the first within 24 hours of admission to the intensive care unit, and then 72 hours thereafter or at the time of recovery, whichever was earlier. The time of recovery was defined as the time when the patient was weaned off all inotropic or vasopressor support. For the control group of patients, a one-time transthoracic echocardiography was performed within 24 hours of diagnosis of sepsis.

Bedside two-dimensional transthoracic echocardiography was performed using a commercially available system, the General Electric Healthcare Vivid q cardiovascular ultrasound system. All examinations were performed by a single operator – a certified critical care specialist with interest in hemodynamics and echocardiography, under the supervision of a certified cardiologist. Echocardiography was performed with the patient in the supine or left lateral position. Images were obtained using a 3.5-MHz ultrasound transducer probe and stored digitally in cine-loop format.

Standard echocardiographic measurements were obtained according to current recommendations by the American Society of Echocardiography [8, 12]. Standard measurements of left ventricular function, including left ventricular ejection fraction (LVEF) and left ventricular index of myocardial performance (LIMP) were obtained. LVEF and left ventricular volumes were measured with the biplane method of disk summation (modified Simpson’s rule). LIMP and right ventricular index of myocardial performance (RIMP) were calculated from pulsed wave Doppler measurements of the isovolumetric contraction, isovolumetric relaxation, and ejection times.

Cardiac output was calculated by the velocity time integral (VTI) measured by Doppler echocardiography at the left ventricular outflow tract (LVOT). The cardiac preload was represented by right atrial pressure, estimated by inferior vena cava (IVC) diameter and the presence of inspiratory collapse. The cardiac afterload, namely systemic vascular resistance, was calculated as the difference between the mean arterial pressure and central venous pressure (or right atrial pressure) divided by the cardiac output.

Two-dimensional speckle tracking echocardiography (STE) was assessed along the longitudinal coordinate of the left ventricle. The left ventricular global longitudinal strain (GLS) was obtained by averaging values obtained in the longitudinal three-chamber, two-chamber, and four-chamber planes in accordance with current guidelines [8]. Strain is defined as the change in myocardial fiber length (end-systole minus end-diastole) as a percentage of the end-diastole myocardial fiber length. Online analysis of strain was performed at the time of image acquisition; repeat analysis was performed offline by a single blinded operator who is a certified cardiologist, and the two values obtained were averaged.

### Statistical analysis

Results were analyzed with SPSS Statistics version 21 for Mac (IBM Corp). We report the number and percentage for categorical variables, and the mean and standard deviation for continuous variables with normal distribution. Categorical variables were compared using the Fisher exact test or Pearson chi-square test, and continuous variables were compared using the Mann-Whitney *U* test or Student *t* test. All tests of significance were two-tailed and a *P* value of 0.05 was considered to be statistically significant.

## Results

### Recruitment

**Fig. 1:**
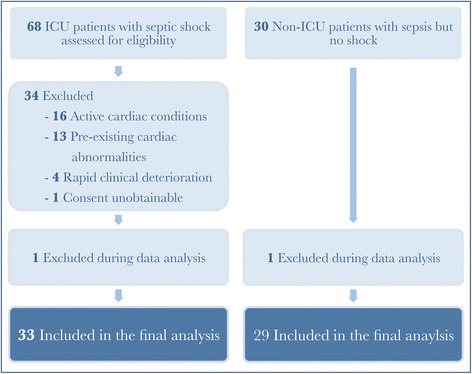
Flow diagram of the study. There were 16 patients excluded due to active cardiac conditions, including 4 patients with tachyarrhythmia, 4 patients on veno-arterial extracorporeal membrane oxygenation support, 3 patients with takotsubo cardiomyopathy, 2 patients were post-resuscitation for cardiac arrest, and 1 patient each with ST elevation myocardial infarction, myocarditis, and pericardial effusion. There were 13 patients excluded based on preexisting cardiac abnormalities, including 5 patients with ischemic cardiomyopathy, 3 patients with dilated cardiomyopathy, 2 with severe aortic stenosis, and 1 patient each with thyrotoxic heart disease, cardiac amyloidosis, and congenital heart disease

From 1 January 2014 through 31 January 2015, 68 consecutive adult medical patients were admitted to the intensive care unit with a diagnosis of septic shock and considered for selection: 16 patients were excluded due to active cardiac conditions, 13 patients were excluded based on preexisting cardiac abnormalities, 4 patients had rapid clinical deterioration and died before complete echocardiographic examination was possible. One patient was excluded because informed consent could not be obtained, and one patient was excluded during echocardiographic data processing due to unsatisfactory image quality. Details of the excluded patients are given in Fig. 1.

The control group were recruited retrospectively, and consisted of 30 patients who were admitted to the medical ward with sepsis but did not develop septic shock. One patient was excluded due to unsatisfactory echocardiographic image quality.

### Patient characteristics

Key clinical characteristics of the patients recruited into the study are shown in Table 1. Baseline characteristics did not differ significantly between the study and control groups. The mean ages were 62 years and 58 years respectively, and 36.4 % vs 41.4 % were women. The two groups were well-balanced with regards to major cardiovascular risk factors and medication use at baseline. The study group had a lower mean Glasgow coma scale (GCS) (11.8 vs 15, *P* < 0.001), higher incidence of bacteremia (45.5 % vs 10.7 %, *P* = 0.027), and greater need for renal replacement therapy (12.1 % vs 0 %, *P* = 0.053) and mechanical ventilation (51.5 % vs 0 %, *P* < 0.001). The mean SOFA score for the study and control groups was 10.2 and 1.6 respectively (*P* < 0.001).

**Table 1:** Baseline characteristics of the patient population

	Study group (*n* = 33)	Control group (*n* = 29)	*P* value
Age, mean (years)	62.2	57.9	0.367
Female sex	12 (36.4 %)	12 (41.4 %)	0.686
Body mass index (kg/m^2^), mean	22.3	22.1	0.826
Hypertension	11 (33.3 %)	8 (27.6 %)	0.624
Diabetes mellitus	8 (24.2 %)	6 (20.7 %)	0.739
Ischemic heart disease	0 (0 %)	1 (3.4 %)	0.282
Chronic renal failure	3 (9.1 %)	2(6.9 %)	0.752
Angiotensin converting enzyme inhibitor	5 (15.2 %)	4 (13.8 %)	0.880
Angiotensin receptor blocker	0 (0 %)	1 (3.4 %)	0.282
Beta blocker	6 (18.2 %)	1 (3.4 %)	0.067
Calcium channel blocker	7 (21.2 %)	3 (10.3 %)	0.246
Diuretic	1 (3 %)	1 (3.4 %)	0.926
Statin	2 (6.1 %)	4 (13.8 %)	0.304
Oxygen saturation, %	97.7 %	97.7 %	0.919
Body temperature (^o^C), mean	37.3	37.5	0.195
Glasgow coma scale, mean score	11.8	15	<0.001*
Bacteremia	15 (45.5 %)	3 (10.7 %)	0.027*
- Gram positive	2 (6.1 %)	0 (0 %)	
- Gram negative	12 (36.4 %)	3 (10.7 %)	
- Others	1 (3 %)	0 (0 %)	
Renal replacement therapy	4 (12.1 %)	0 (0 %)	0.053*
Mechanical ventilation	17 (51.5 %)	0 (0 %)	<0.001*
- Non-invasive ventilation	1 (3 %)	0 (0 %)	
- Invasive mechanical ventilation	16 (48.5 %)	0 (0 %)	
SOFA score, mean	10.2	1.6	<0.001*

Results are presented as number of patients (percentage within group in parentheses) unless stated otherwise. *SOFA* sequential organ failure assessment. *P < 0.05

The mean duration of vasopressor or inotropic therapy for the study group was 80.4 hours (median 42 hours, interquartile range 23–62.5 hours). Amongst the 33 patients in the study group, the 30-day all-cause mortality rate was 18.2 % (*n* = 6), and the 90-day all-cause mortality rate was 41.9 % (*n* = 13). There were no deaths in the control group (Table 2).

**Table 2:** Clinical outcomes

	Study group	Control group	*P* value
30-day mortality	6 (18.2 %)	0 (0 %)	0.034*
90-day mortality	13 (41.9 %)	0 (0 %)	0.002*
Length of ICU stay, mean (days)^a^	12.5	N/A	N/A
Length of hospital stay, mean (days)^b^	55.2	5.2	0.001*
Duration of vasopressors, mean (hours)^c^	80.4	0	<0.001*

Results are presented as number of patients (percentage within group in parentheses) or mean. ^a^Length of ICU stay, median 6 days, interquartile range 10 days. ^b^Length of hospital stay, median 28 days, interquartile range 48 days. ^c^Duration of vasopressor use, median 42 hours, interquartile range 40 hours. *P < 0.05

*NA* not applicable

### Hemodynamic data

Bedside hemodynamic vital signs were different amongst the two groups of patients. The study group had a lower mean arterial pressure (76 mmHg vs 82 mmHg, *P* = 0.032), and a higher heart rate (99 bpm vs 86 bpm, *P* = 0.008). Measured by echocardiography, the study and control groups had a similar mean stroke volume (59.6 mL vs 61.7 mL, *P* = 0.620), mean cardiac output (5.88 L/min vs 5.48 L/min, *P* = 0.401), and mean cardiac index (3.48 L/min/m^2^ vs 3.34 L/min/m^2^, *P* = 0.608) (Table 3).

**Table 3:** Mean standard two-dimensional echocardiographic measurements

	Study group	Control group	*P* value
Left heart volume			
LAD (cm)	3.12	3.06	0.601
LVEDV (mL)	74.09	74.69	0.901
LVESV (mL)	30.21	29.00	0.591
Left ventricular function			
LVEF (%)	59	61	0.169
FS (%)	32	33	0.163
LIMP	0.25	0.22	0.319
Right ventricular function			
RIMP	0.25	0.18	0.083
TAPSE (cm)	1.82	2.16	0.001*
RVSP (mmHg)	30.87	25.30	0.054
Hemodynamic data			
MAP (mmHg)	76	82	0.032*
Heart rate (bpm)	99	86	0.008*
Stroke volume (mL)	59.6	61.7	0.620
Cardiac output (L/min)	5.88	5.48	0.401
Cardiac index (L/min/m^2^)	3.48	3.34	0.608
RAP (mmHg)	7.4	5.9	0.017*
SVR (dynes•sec/cm^5^)	1090	1194	0.303
SVRI (dynes•sec/cm^5^/m^2^)	1807	1976	0.333

*Abbreviations*: *LAD* left atrial diameter, *LVEDV* left ventricular end-diastolic volume, *LVESV* left ventricular end systolic volume, *LVEF* left ventricular ejection fracture, *FS* fractional shortening, *LIMP* left ventricular index of myocardial performance, *RIMP* right ventricular index of myocardial performance, *TAPSE* tricuspid annular plane systolic excursion, *RVSP* right ventricular systolic pressure, *MAP* mean arterial pressure, *RAP* right atrial pressure, *SVR* systemic vascular resistance, *SVRI* systemic vascular resistance index. *P < 0.05

The cardiac preload, represented by right atrial pressure, was higher in the study group (7.4 mmHg vs 5.9 mmHg, *P* = 0.017). The afterload was similar (systemic vascular resistance 1090 dynes•sec/cm^5^ vs 1194 dynes•sec/cm^5^, *P* = 0.303; systemic vascular resistance index 1807 dynes•sec/cm^5^/m^2^ vs 1976 dynes•sec/cm^5^/m^2^, *P* = 0.333).

### Conventional echocardiographic measurements

**Fig. 2:**
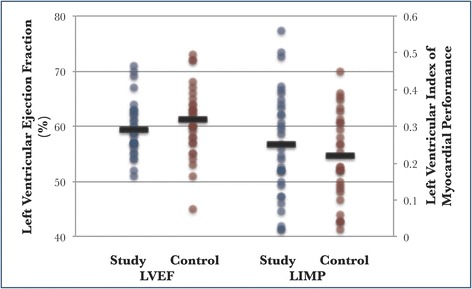
Standard echocardiographic indices of left ventricular function. Left ventricular ejection fraction (*LVEF*): mean 59 % vs 61 %, *P* = 0.169. Left ventricular index of myocardial performance (*LIMP*): mean 0.25 vs 0.22, *P* = 0.319

There were no statistically significant differences between the study group and control group in any measures of left ventricular function. These include the LVEF (59 % vs 61 %, *P* = 0.169), fractional shortening (FS) (32 % vs 33 %, *P* = 0.163), and left ventricular index of myocardial performance (LIMP) (0.25 vs 0.22, *P* = 0.319) (Fig. 2).

### Strain measurements

**Fig. 3:**
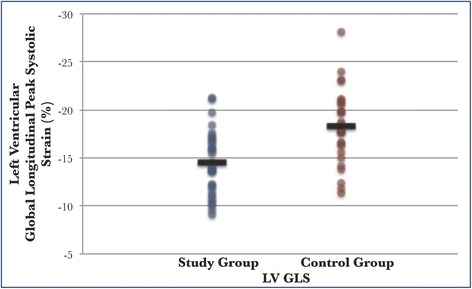
Left ventricular global longitudinal strain in the study and control groups at diagnosis of sepsis. Left ventricular global longitudinal peak systolic strain (*LV GLS*): mean –14.5 % vs –18.3 %, *P* < 0.001

The study group had a greater degree of myocardial dysfunction measured by left ventricular global longitudinal strain (GLS) (–14.5 % vs –18.3 %, *P* < 0.001) (Fig. 3). The difference in strain persisted across segmental strain values obtained at the left ventricular three-chamber (–14.1 % vs –18.6 %, *P* < 0.001), two-chamber (–13.5 % vs –18.3 %, *P* < 0.001), and four-chamber measurements (–14.7 % vs –17.9 %, *P* = 0.002) (Table 4).

**Table 4:** Mean strain values in the study and control groups at diagnosis of sepsis

	Study group	Control group	*P* value
GLS	–14.46 %	–18.25 %	<0.001*
Longitudinal three-chamber strain	–14.10 %	–18.56 %	<0.001*
Longitudinal two-chamber strain	–13.53 %	–18.29 %	<0.001*
Longitudinal four-chamber strain	–14.72 %	–17.90 %	0.002*

*P < 0.05

*GLS* Global longitudinal strain

On comparing the first and second echocardiographic examinations in the study group who could be weaned off vasopressors or inotropes (*n* = 23), there was a significant improvement in left ventricular strain upon recovery (–14.6 % vs –16.0 %, *P* = 0.026). In nonsurvivors, this difference in GLS was not detected (–15.3 % vs –15.8 %, *P* = 0.563) (Table 5).

**Table 5:** Mean strain values in the study group at diagnosis of and recovery from septic shock

	Diagnosis	Recovery	*P* value
Study group (*n* = 33)	–14.46 %	–16.02 %	0.010*
Patients weaned off vasopressors (*n* = 23)	–14.57 %	–15.97 %	0.026*
Patients not weaned off vasopressors (*n* = 10)	–14.19 %	–16.15 %	0.194
Nonsurvivors (*n* = 13)	–15.28 %	–15.79 %	0.563

*P < 0.05

## Discussion

This is one of the first case-control studies to apply the relatively novel technology of STE in the diagnosis and monitoring of sepsis-induced myocardial depression in the adult population. There are two important conclusions. The first part of our study involved comparing the strain values in patients with septic shock and patients with sepsis only. We observed a significant difference in the measured GLS (–14.5 % vs –18.3 %, *P* < 0.001) between the two groups of patients. The strain value of –18.3 % in our control group is comparable to the previously reported range of –17 % to –23 % in normal healthy subjects [13, 14]. This is in contrast to patients with septic shock, where myocardial impairment is present, evidenced by the depressed strain value of –14.5 % in our study group. Importantly, conventional echocardiographic parameters of left ventricular function, including LVEF, FS, and LIMP, all failed to detect a difference between the two groups of patients. Our data mirror the findings from a pediatric population, reported by Basu et al. [15], where the GLS was –14.4 % in the septic group and –23.3 % in the control group (*P* < 0.001). Again, there was no significant difference in LVEF and FS between the two groups of patients.

Recent research has provided preliminary data indicating that strain may be sensitive to acute changes in load [16]. We undertook efforts to minimize the interplay of other hemodynamic variables on the measurement of cardiac strain. The cardiac preload in the study group was higher (7.4 mmHg vs 5.9 mmHg, *P* = 0.017), by a negligible absolute value of 1.5 mmHg, evidencing that these patients with hypotension have been adequately volume-resuscitated prior to echocardiographic analysis. All patients in the study group were instituted on vasopressors, with comparable afterload indices between the two groups (systemic vascular resistance 1090 dynes•sec/cm^5^ vs 1194 dynes•sec/cm^5^, *P* = 0.303; systemic vascular resistance index 1807 dynes•sec/cm^5^/m^2^ vs 1976 dynes•sec/cm^5^/m^2^, *P* = 0.333).

The second part of this study involved serial echocardiographic examinations in the group of patients with septic shock. In the patients who could be weaned off vasopressors by 72 hours, we observed a difference in strain values obtained at diagnosis and recovery (–14.6 % vs –16.0 %, *P* = 0.026). Nonsurvivors did not exhibit the improvement in myocardial strain at 72 hours (–15.3 % vs –15.8 %, *P* = 0.563). This supports the current understanding of septic cardiomyopathy as a reversible form of myocardial impairment during the sepsis syndrome.

The diagnosis of sepsis-induced myocardial impairment has been difficult without the availability of a sensitive and specific bedside diagnostic tool. One of the commonest measures of left ventricular function, LVEF, is affected to a significant degree by the changing preload and afterload in sepsis. Vieillard-Baron, in a series of echocardiography studies, showed that when performed at different times after the onset of septic shock, echocardiography yields different incidences of left ventricular dysfunction [17, 18]. In the first 6 hours of resuscitation, there was an 18 % incidence of decreased left ventricular ejection fraction; this rose to 40 % after 24 hours, and 60 % after 2–3 days. The postulation to explain this phenomenon was the increase in afterload with time, secondary to the vasopressors used for resuscitation, or the natural resolution of the disease. The authors concluded, in a review on septic cardiomyopathy [19], that the measurement of LVEF “actually reflects the (left ventricular) afterload rather than the intrinsic contractility”.

STE has emerged as a direct, angle-independent, and highly reproducible measurement of left ventricular function. Its application in conditions such as heart failure with preserved ejection fraction [20] has shown it to offer incremental and prognostic value in the assessment of left ventricular performance. GLS is the most commonly used strain-based measure of left ventricular global systolic function, and its use has been included in international echocardiography guidelines [8]. With the development in software technology, basic strain measurements may now be performed at the bedside, and our experience has shown it to be feasible with patients in septic shock admitted to the intensive care unit.

We managed to show how STE may be useful as a tool for diagnosis as well as disease monitoring in sepsis-induced myocardial impairment. It enables the detection of subtle left ventricular dysfunction early in the course of illness, which is not otherwise detected by measurement of LVEF alone, and further discriminates the reversibility of this condition in patients who subsequently recover. In demonstrating similar preload and afterload parameters in our study and control groups, we have eliminated possible interference by these factors in the measurement of strain. The proper identification and description of sepsis-induced myocardial impairment may have important therapeutic implications, guiding the use of cardioprotective strategies such as β-blockers in the management of patients with septic shock [21].

Our study has several limitations. First, the sample size was small, especially in the subgroup analyses. Second, as the timing of the reassessment echocardiogram as either when the patient came off inotropic or vasopressor support, or at 72 hours, was decided arbitrarily, we were unable to prove the complete reversibility of strain measurements to normal values upon recovery. Third, we did not have a resuscitation protocol for the choice and dose of inotropes and vasopressors. Finally, our data were not compared to more objective modalities of quantifying ventricular function, such as cardiovascular magnetic resonance imaging or radionuclide ventriculography.

## Conclusion

In conclusion, sepsis-induced myocardial impairment can be detected by left ventricular global longitudinal strain measured by speckle tracking echocardiography in patients with septic shock, and this was not otherwise identifiable by conventional echocardiographic parameters. Strain measurement, which can now be performed at the beside, is useful for the diagnosis and monitoring of this disease. Further studies are needed to define cutoff values and evaluate its application as a potential diagnostic tool for sepsis-induced myocardial depression.

## Key messages

- Strain measurements are more sensitive than conventional echocardiographic parameters in detecting sepsis-induced myocardial impairment.
- Patients with septic shock have a greater degree of myocardial impairment compared to patients with sepsis only.
- Strain measurements can feasibly be performed at the bedside and can be used for serial monitoring of sepsis-induced myocardial impairment.

## Abbreviations

FS: fractional shortening
GCS: Glasgow coma scale
GLS: global longitudinal strain
LIMP: left ventricular index of myocardial performance
LVEF: left ventricular ejection fraction
LVOT: left ventricular outflow tract
MPI: myocardial performance index
RIMP: right ventricular index of myocardial performance
SOFA score: sequential organ failure assessment score
STE: speckle tracking echocardiography
SVR: systemic vascular resistance
SVRI: systemic vascular resistance index
TDI: tissue Doppler imaging
VTI: velocity time integral
